# Exploring built environment factors on e-bike travel behavior in urban China: A case study of Jinan

**DOI:** 10.3389/fpubh.2022.1013421

**Published:** 2022-09-12

**Authors:** Yonghao Yu, Yuxiao Jiang, Ning Qiu, Heng Guo, Xinyu Han, Yuanyuan Guo

**Affiliations:** ^1^Department of Architecture and Urban Planning, Shandong Jianzhu University, Jinan, China; ^2^School of Architecture, Tianjin University, Tianjin, China; ^3^Public Planning Center, Jinan City Planning and Design Institute, Jinan, China

**Keywords:** built environment, e-bike usage, multi-source data, geographical weighted regression, LISA, spatial heterogeneity, Jinan

## Abstract

E-bike, characterized as a low-carbon and health-beneficial active travel mode, is gradually becoming popular in China. Although built environment factors are considered to be key parameters that can facilitate or hinder active transportation, such as cycling or walking, few studies have explored the impact of built environment on e-bikes. To fill this gap, this study was the first to explore the relationship between e-bike usage and built environment factors based on population level travel survey in central Jinan, China. Both macro and micro levels of built environment were measured using multi-source data. We employed ordinary least squares (OLS) and geographically weighted regression (GWR) models to explore the aggregation patterns of e-bike trips. Besides, the local Moran's I was employed to classify the aggregation patterns of e-bike trips into four types. The results from OLS model showed that eye-level greenery, building floor area, road density and public service POI were positive significantly related to e-bike trips, while open sky index and NDVI had negative association with e-bike trips. The usage of GWR model provided more subtle results, which revealed significant spatial heterogeneity on the impacts of different built environment parameters. Road density and public service POI posed positive effects on e-bike travel while NDVI and open sky index were found mainly pose negative impacts on e-bike travel. Moreover, we found similar coefficient distribution patterns of eye-level greenery, building floor area and distance to bus stop. Therefore, tailored planning interventions and policies can be developed to facilitate e-bike travel and promote individual's health level.

## Introduction

### The prevalence of e-bike and its advantages

For the past few years, the built environment which is suitable for active transportation have attracted interests from various of fields including urban design, public health and transportation. Global economic development has been driven by the fossil energy consumption which improved human living conditions but caused air pollution and global warming and a series of environmental problems (1). Energy-saving, low-carbon, healthy lifestyle have become the common will of the government and citizens (2). Governments have implemented policies including improving energy efficiency, developing fossil energy substitution technologies, and using biocarbon sink technologies to reduce carbon emissions (3). In addition, it is a key intervention to maintain physical activity level and reduce chronic disease by encouraging residents to adopt active transportation (i.e., walking and cycling, e-bike) rather than apply motorized vehicles (4). Therefore, it is vital to understand the characteristics of active transportation and its driving factors for planning department and policy makers (5).

As an emerging active transportation mode, the advantages of e-bike are embodied in several ways. First, compared to traditional cycling, e-bike is time efficient (6) and aging-friendly which can help users to overcome obstacles of long distance and climbing (7). Meanwhile, e-bike travel also combines the advantages of promoting personal physical fitness and well-being (8). Specifically, e-bike travel can benefit those who are unwilling to engage in physical activity (i.e., overweight, disabled, and elderly) by helping them achieve moderate vigorous of physical activity (9, 10), which is known to support individual's social interaction (11) and mental health (8). Some studies have shown that e-bike travel improves metabolism (12), cardiovascular health (13) and allows riders to have lower level of perceived exercise and higher level of enjoyment (14). Moreover, e-bike is more economically efficient with lower purchase price, less operating and maintenance cost than automobile, thus is becoming a competitive alternative (15). In addition, some studies have shown that using an e-bike can reduce CO_2_ emission by about 460 kg per year, which is an impressive social benefit (16).

The numerous benefits of e-bikes have made them popular with residents in many countries. It is expected that more than 40 million e-bikes will be sold worldwide in 2023 generating approximately 20 billion dollars in revenue (17). In some low-density Western countries such as Italy, Germany and Canada, residents ride e-bikes primarily for leisure and exercise (18). These countries often have a unique cycling culture, with sophisticated cycling facilities and fewer safety barriers. In the last few years, several countries, e.g., Belgium, Norway, Netherlands, have started to subsidize the purchase of e-bikes to support e-bike assisted commuting (19). Furthermore, majority cities in Asia are still in the rapid development stage. The high-density urban environment and high-intensity economic pressure have raised the great demand for e-bikes. According to statistics, the sales of e-bikes in countries including China, Vietnam and Japan are rising year by year, occupying more than half of the global market share (20). China, in particular, is a global leader in the manufacture of e-bikes as well as in the annual and total number of e-bikes sales. By the end of 2019, China had over 300 million e-bikes and the market size reached trillions (21). Rising gasoline price, declining e-bike technology cost and deteriorating road congestion and parking problem are the main reasons for the tremendous demands of e-bike (22). As housing price continually rises and the imbalance between job-housing relationship increases, residents' commuting distance has increased, thus e-bike is gradually regarded as a comfortable and efficient way for daily transportation (23).

### Relationship between built environment and e-bike usage

Built environment is usually defined as man-made buildings and places which involve physical facilities as well as abstract elements such as human spatial perception (24). Built environment is measured in various ways. The macro-level built environment was first characterized by 3-Ds element (3Ds), namely density, design and diversity (25). Later studies added distance to public transportation and destination accessibility to form the 5Ds assessment framework (26). In addition, micro-level factors such as open sky, buildings, and greenery visibility perceived by residents were also included in the studies related to built environment (27).

Active travel behavior, as a fundamental travel mode of daily life, has been proven to have strong correlation with built environment elements. For example, proximity to commercial facility and low noise level were prove to be benefit for residents' walking and cycling (28). Pronounced building density and mixed land use had a positive influence on promoting active travel instead of automobile travel (29). Moreover, more residents would choose to travel by bicycle in the areas with high road density, proximity to green space and abundant bicycle parking facilities (30).

Currently, few studies have been conducted by scholars on e-bikes in relation to the built environment. Some studies showed that e-bike is an alternative vehicle in many Chinese cities (31). An empirical study revealed a non-linear association between built environment characteristics and e-bike holdings (32). E-bike holding has negative correlation to residence density while is positively related to distance to public transportation (32). The e-bike usage in China was different among various urban scales. Longer commuting distance in metropolis made e-bike less competitive that public transportation, while e-bike travel was found to be more attractive in middle-size cities (33). In addition, some studies showed that the travel patterns of residents in the rural area differ from those of urban residents. Certain road level (i.e., major trunk road or city road without bike lane) have positive effects in facilitating e-bike usage for rural residents (34).

### Multi-source urban big data to assess built environments

Urban information collection plays a vital role in urban studies and travel behavior research. In the past, traditional urban research has long been constrained by field observation characterized as time-consumption, inefficiency, and small simple size (35). With the rapid development of big data, studies on built environment and travel behavior broke through the limitations of traditional data by applying emerging technologies which can obtain real-time and accurate data to quantify complex built environment factors. New urban data, such as social media data (36), heatmap (37), street view image (38), point of interest (POI) (39) and building footprint data (40), provide urban researchers with fine spatial and temporal granularity. However, the plenty advantages of urban big data cannot completely replace traditional data in some research. Urban big data and traditional data are complementary and the integration of the two is believed to be an inevitable trend (41). Currently, scholars are actively exploring methodologies and specific frameworks for urban research that combine traditional data with big data for the integration of data with various sources in city studies (42, 43).

### Research gap and our study

Despite many studies exploring the relationship between traditional active transportation (i.e., walking, cycling) and the built environment, e-bike related studies were rarely emphasized (44, 45). Previous studies on e-bike travel mainly focused on the personal perception and travel preference of e-bike users (22, 46). Since e-bike is becoming a vital active travel mode in developing countries, clarifying the association between built environment and e-bike usage will enrich active transportation research and assist urban planners in developing more appropriate planning interventions.

In order to eliminate the research gap, e-bike usage was measured using 2019 residential travel characteristics survey in Jinan, China. We applied ordinary least squares (OLS) model, geographically weighted regression (GWR) and Moran's I to reveal the spatial distribution pattern of e-bike usage and the relationship between built environment factors and e-bike usage. This study makes contribution to the existing research from three aspects: (1) Using multi-source data, i.e., street view images and point-of-interest (POI) data to assess macro and micro built environment factors and expand 5D built environment framework. (2) Revealing the global and local effects of built environment on e-bike travel using OLS and GWR models. (3) To our knowledge, this study is among the first articles that attempts to reveal the mechanism of e-bike travel.

## Materials and methods

### Study area and spatial unit

Jinan, the capital of Shandong Province, is a mega-city with a long history. In the past 70 years, Jinan has developed rapidly with the built-up area expanding to 841.2 km^2^ and the resident population growing to 9.336 million. Currently, Jinan has 12 county-level administrative districts (47). However, rapid urbanization has brought many problems to Jinan. The increasing number of urban motor vehicles aggravates the traffic pressure and exacerbates air pollution which has become a major source of urban environmental degradation. In 2020, Jinan ranked as the most congested city in China and the 11th most polluted city in terms of air pollution (48, 49). The drawbacks of motor vehicles have led to a surge in the number of e-bikes in recent years. According to the statistics, about 30.56% of Jinan residents choose e-bikes as their daily travel mode and there are more than 3.6 million e-bikes in Jinan and the number still grows (50). The growing demand for e-bikes places higher requirements on Jinan's future urban construction. Therefore, to unveil the relationship between urban built environment and e-bike travel can help to plan and build a better city.

**Figure 1:**
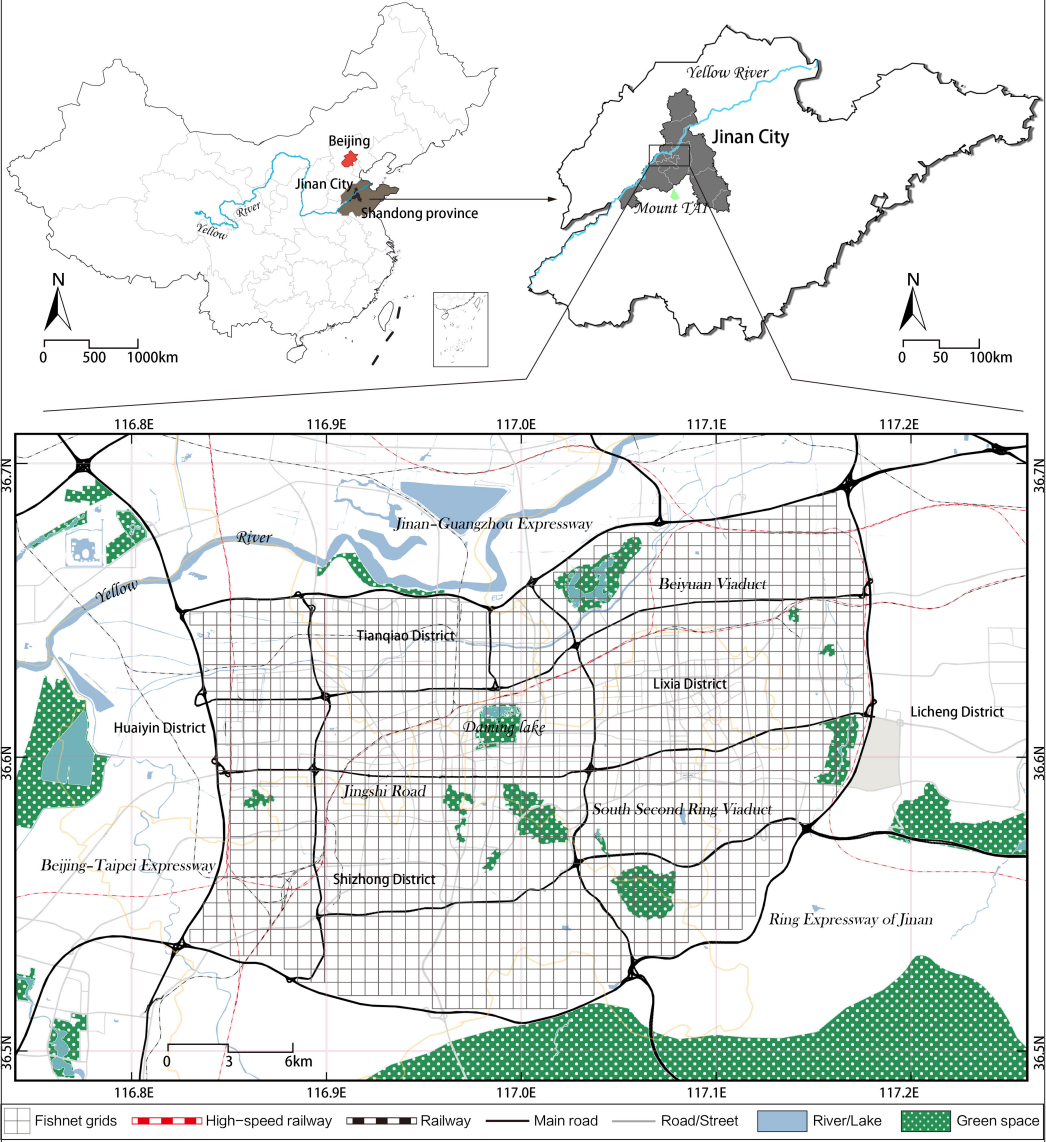
Location of the study area.

The study area includes the central urban area of Jinan (116°51′36″-117°12′25″E, 36°32′51″-36°46′5″N) enclosed by the Jiguang Expressway, the Jingtai Expressway and the Jinan Bypass Expressway, with an area of 535.96 km^2^ (Figure 1). Our study area is located at the central area of Jinan's master plan, and it serves as the core of Jinan's economic, political and cultural center. In the main urban area of Jinan, e-bikes as a flexible mode of travel play as a vital part in people's daily life. This study intends to unveil the relationship between built environment and e-bike usage in Jinan city.

With the increasing attention on active travel in various countries, the government is gradually promoting the construction of 15-minute city (51), where 500–800 m is considered proximity to neighborhood residents' activity and is therefore widely applied in walking- or cycling-related studies (52). In the study area, a 600 × 600 m rectangular grid was generated as the basic analytical unit which can facilitate the integration of urban built environment statistics with e-bike usage data (Table 1), and help to eliminate the effect of uneven administrative division (53). Then, the data related to e-bike travel behavior and urban built environment were processed and imported into the mapping and analysis software ArcGIS (version 10.6) for geocoding, among which the grids that did not contain e-bike travel data were removed, leaving a total of 770 grids in the study area.

**Table 1:** Definitions of the dependent and independent variables.

**Variables (unit)**	**Definition**
**Dependent variable**	
Number of E-bike trips (N)	The total amount of e-bike trips (destination or origination) in each grid
**Independent variable**	
**Micro scale built environment**	
Eye-level greenery	The average ratio of greenery of all SVIs in each grid
Open sky index	The average ratio of open sky of all SVIs in each grid
**Macro scale built environment**	
Building floor area (m^2^)	The total building floor areas in each grid
Land-use mix (≥0)	The ratio of different land-use types in each grid
Road density (m)	The total road length (m) in each grid
Commercial POI (N)	The number of corresponding POIs in each grid (acquired from http://map.baidu.com)
Public service POI (N)	
Distance to bus stop (m)	The distance from the nearest bus stop in each grid
NDVI	The average NDVI value of each grid

### Data source and variables

#### Dependant variable

In this study, e-bike travel behavior data were obtained from the Jinan Resident's Travel Survey of 2019 (JNRTS 2019), which was conducted to study the travel behavior of Jinan citizens. In order to ensure the completeness and representativeness of the sample, the JNRTS 2019 was conducted in July 2019 by trained interviewers followed a proportional to population size (PPS) method to obtain the sample. Respondents were requested to offer one-day elaborate travel records. Taking e-bike travel as an example, respondents were asked to record personal travel message such as the start and end times, initial and final locations. In total, the survey recruited 44,084 adults from 698 communities in central Jinan. All locations of e-bike trips were geocoded and relocated in corresponding fishnets adopting ArcGIS 10.6. At last, the number of e-bike trips within a gird was used as the dependent variable.

#### Macro-level built environment features

The independent variables in this study, macro-level built environment factors, were assessed according to 5Ds framework, including density, design, diversity, destination accessibility and distance to public transportation (26). In addition, numerous studies have shown that urban greenery has a significant impact on active travel (54).

Density is measured based on the total building floor areas in every grid. Diversity is calculated by using land-use mix of five fundamental point of interest (POI) categories (i.e., commercial, residential, public service, tourism and education) in each grid (55). Land-use mix is calculated as follows:


$$\begin{array}{l} E_{j}=\frac{-\sum_{i}^{\;}\left(A_{ij}\ln\left(A_{ij}\right)\right)}{\ln\left(N_{j}\right)} \end{array}$$

Where *A*_*ij*_ indicates the percentage of POIs of category i in grid j; *N*_*j*_ is the number of POI types in grid j.

Design is measured based on road density, namely the total road length (m) in each grid. We applied the number of POIs in each grid as destination accessibility. Distance to public transportation is calculated by the shortest physical distance to bus stop (56).

Finally, we employed normalized difference vegetation index (NDVI), a commonly used parameter for vegetation assessment, as indicator of urban greenery (57). NDVI is calculated by Landsat-8 remote-sensing image acquired in June 2018, the calculation formula is shown as follows:


$$\begin{array}{l} NDVI=\frac{NIR-Red}{NIR+Red} \end{array}$$

Where Red and NIR denote spectral reflectance measurements extracted from red and near infrared areas, respectively. The values of NDVI are between 0 to 1, a high NDVI value suggest a high level of vegetation.

#### Micro-level built environment features

**Figure 2:**
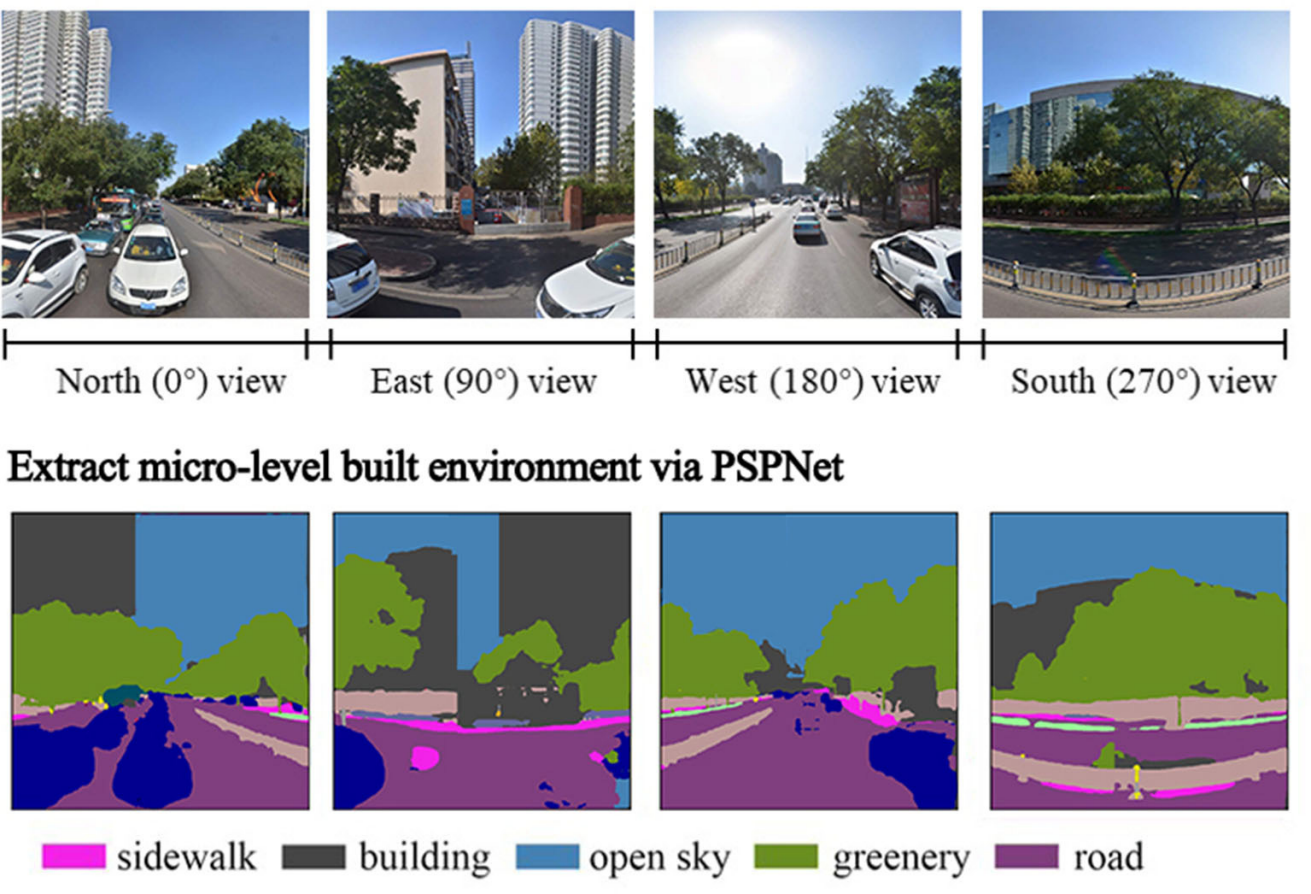
Assessing micro-level built environment from Baidu street view image *via* machine learning Technique (PSPNet).

We collected the pedestrian's eye-level streetscape features *via* street view image (SVI) as the micro-scale built environment characteristics (58). Sampling points were obtained every 50 meters along the urban roads by Open Street Map (OSM). Then four SVIs (1,024^*^1,024 pixels) with a 90° field of view were collected for each sampling point through Baidu Maps' API (https://lbsyun.baidu.com/). The heading parameters 0°, 90°, 180°, and 270° of the four pictures collected at each sampling site representing north, east, south, and west, respectively. In this study, 519,388 street view images were retrieved from 129,847 sampling sites in Jinan. We then performed a Pyramid Scene Parsing Network (PSPNet) with a Cityscapes model to classify the foreground objects in the image into 19 categories, calculate the pixel percentage of every streetscape feature in the image and finally obtain the average value of each streetscape feature at each sample point. Since the vegetation (eye-level greenery) and open sky index are widely used in active-transportation related studies (59–61), we selected them as micro-level built environment variables, which were measured by calculating the average value in each grid (Figure 2).

### Statistical analysis

**Figure 3:**
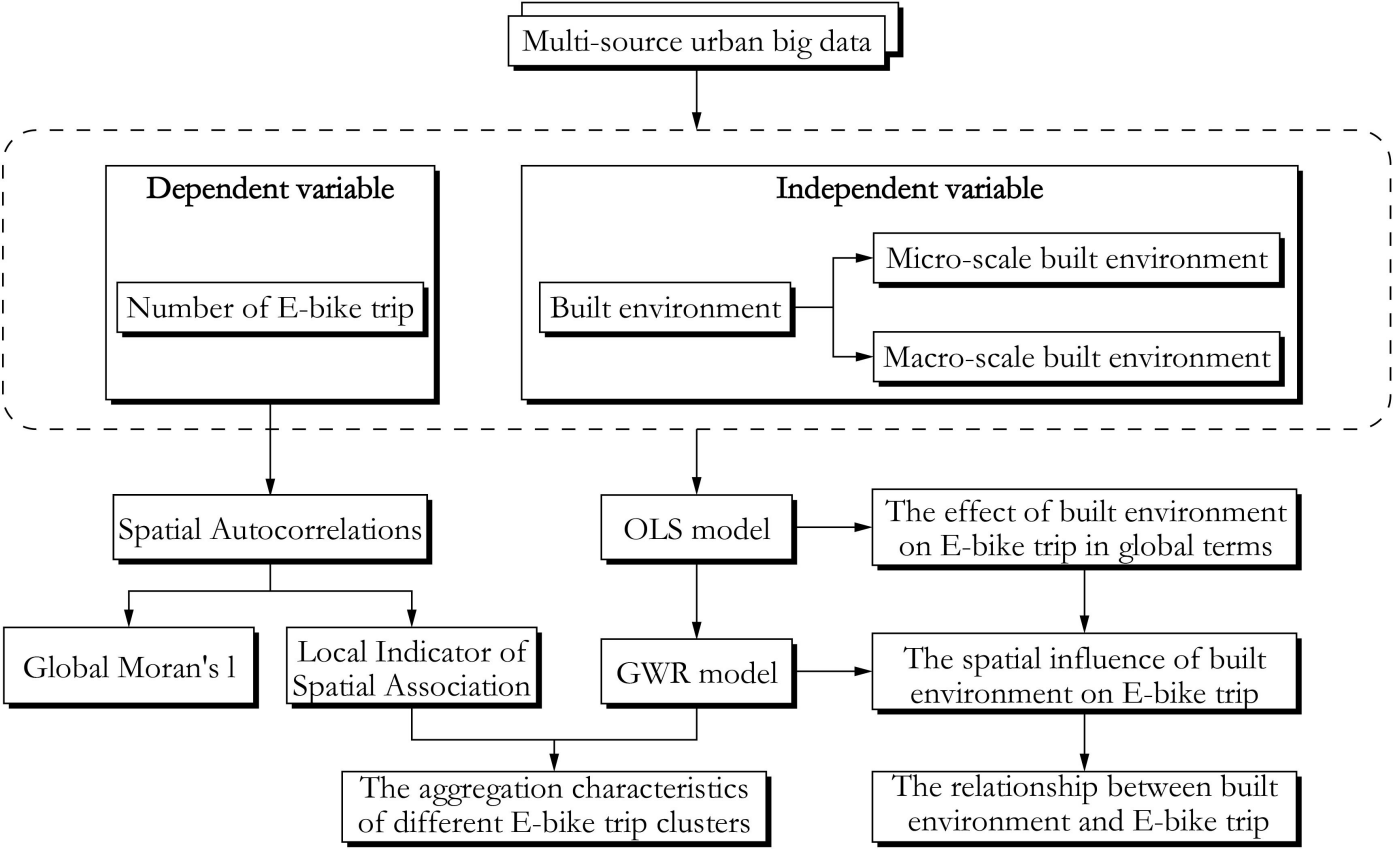
Technology roadmap.

In the research, global Moran's I and local Moran's I were employed to characterize the global and local aggregation pattern of e-bike usage. We first tested the variance inflation factor (VIF) between the independent variables. All variables with VIF >4 were excluded from the subsequent analysis. Thus, education and residential POI were excluded. OLS and GWR model were used to better quantify the built environment elements-e-bike usage association. In addition, we combined the results of the GWR model with Local Indicators of Spatial Association (LISA) to further explore the local aggregation characteristics of e-bike trips (Figure 3).

#### Spatial autocorrelation

Spatial autocorrelation is an essential indicator to examine whether the attribute value of a factor is significantly associated with its value of non-boring unit (62). Global Moran's I indicates the overall distribution of data within the study area, while local Moran's I assesses the similarities and differences between neighboring units (63). Global and local Moran's I are calculated as follows:


$$\begin{array}{l} Global\;Moran^{\prime }s\;I=\frac{n\sum_{i=1}^{n}\sum_{j=1}^{n}\omega_{ij}\left(x_{i}-\bar{x}\right)\left(x_{j}-\bar{x}\right)}{\sum_{i=1}^{n}\sum_{j=1}^{n}\omega_{ij}\sum_{i=1}^{n}{(x_{i}-\bar{x})}^{2}}\\ \;Local\;Moran^{\prime }s\;I=\frac{x_{i}-\bar{x}}{S^{2}}\sum_{j=1}^{n}\omega_{ij}\left(x_{j}-\bar{x}\right)\\ \;S^{2}=\frac{\sum_{j=1,j\neq i}^{n}\omega_{ij}\left(x_{j}-\bar{x}\right)}{\left(n-1\right)}-{\bar{x}}^{2} \end{array}$$

where *x*_*i*_ and *x*_*j*_ denote the total e-bike trips of grid i and j, respectively; n denotes the total count of girds; $\bar{x}$ is the mean e-bike trips of n grids; ω_*ij*_ is the spatial weight matrix. The value of Moran's I is distributed between −1 and 1. When Moran's I index is above 0, it indicates that the attribute values of all girds have positive spatial correlation. While Moran's I index below 0 indicates that the attribute values of all girds have negative spatial correlation.

Local Indicators of Spatial Association (LISA) is a method based on local Moran's I proposed by Anselin, and this method can reveal the possible spatial heterogeneity (64). We applied LISA to distinguish the studies into four types i.e., high-high (H-H), low-low (L-L), high-low (H-L) and low-high (L-H). The H-H type represents high value clustering and the L-L type represents low value clustering. The units of H-L type denote high values surrounded by low values, while the L-H units denote low values surrounded by high values.

#### Regression analysis

In this study, OLS and GWR models were conducted to quantify the relationship between built environment and e-bike travel. The OLS model is a regression model commonly used to analyze linear relationships between variables (65). The OLS model is calculated as follows:


y=Xβ+ε

where y is the e-bike trips, X is the matrix of the independent variable, β is a vector of the coefficient, and ε is a vector of random error term (66).

The GWR model is a further extension of the OLS model, and this model can explore the spatial variation patterns of influencing factors in different geographical locations (67). The spatial relationship among multiple built environment variables can be effectively processed using the GWR model to better explain the variables affecting e-bike travel. The GWR model is expressed as follows:


$$y_{i}=\beta_{0}\left(u_{i},v_{i}\right)+\sum_{k=1}^{\;}\beta_{k}\left(u_{i},v_{i}\right)x_{ik}+\varepsilon_{i}\;i=1,\ldots ,n$$

where (*u*_*i*_, *v*_*i*_) denotes the coordinates of unit i; β_0_(*u*_*i*_, *v*_*i*_) denotes the intercept value; and β_*k*_(*u*_*i*_, *v*_*i*_) is the set of parameter values at unit i. Different from the spatially fixed coefficient of OLS model, GWR model allows the parameter estimates to vary with units and therefore may capture local effects (68).

## Results

### Descriptive statistics

The descriptive statistics of e-bike ridership and built environment are shown in Table 2. The average value of e-bike usage in each grid is 43.30 (SD = 53.980), indicating that there was an average of ~43 e-bike trips occurred in each unit. Besides the count span of e-bike usage between spatial units is substantial, ranging from 1 to 394.

**Table 2:** Statistics for all variables within the Jinan study area, sampled in 2019 (fishnet = 600 × 600 m, *N* = 770).

**Variables (unit)**	**Min**.	**Max**.	**Mean**	**SD**
**Dependent variable**
Number of E-bike trips (N)	1	394	43.300	53.980
**Independent variable**
**Micro scale built environment**
Eye-level greenery	0	0.552	0.147	0.081
Open sky index	0	0.440	0.254	0.081
**Macro scale built environment**
Building floor area (m^2^)	0	197,202.304	63,536.748	43,409.640
Land-use mix (≥0)	0	1.000	0.696	0.344
Road density (m)	0	17,255.150	3,997.850	2,402.737
Commercial POI (N)	0	189	18.030	25.651
Public service POI (N)	0	227	22.740	30.252
Distance to bus stop (m)	0	1,230	56.620	136.592
NDVI	0.061	0.338	0.164	0.047

N, number; Min., minimum; Max., maximum; SD, standard deviation; POI, point of interest; NDVI, normalized difference vegetation index.

For the micro-built environment, the standard deviation (SD) of both open sky index (Mean = 0.254, SD = 0.081) and eye-level greenery (Mean = 0.147, SD = 0.081) were the same. Open sky index had a greater average value than eye-level greenery, which indicated that the proportion of open sky was larger than that of greenery in most spatial units. In terms of macro-built environment, the study area had a relatively high building density (Mean = 63,536.748, SD = 43,409.640) and a highly mixed land use level (Mean = 0.696, SD = 0.344). In addition, the study area owned a well-connected transportation system with a high road density (Mean = 3,997.850, SD = 2,402.737) and convenient proximity of public transit (Mean = 56.620, SD = 136.592). Moreover, the mean value of public service POI (Mean = 22.740, SD = 30.252) was higher than that of commercial POI (Mean = 18.030, SD = 25.651).

### Spatial distribution pattern of e-bike travel volume

**Figure 4:**
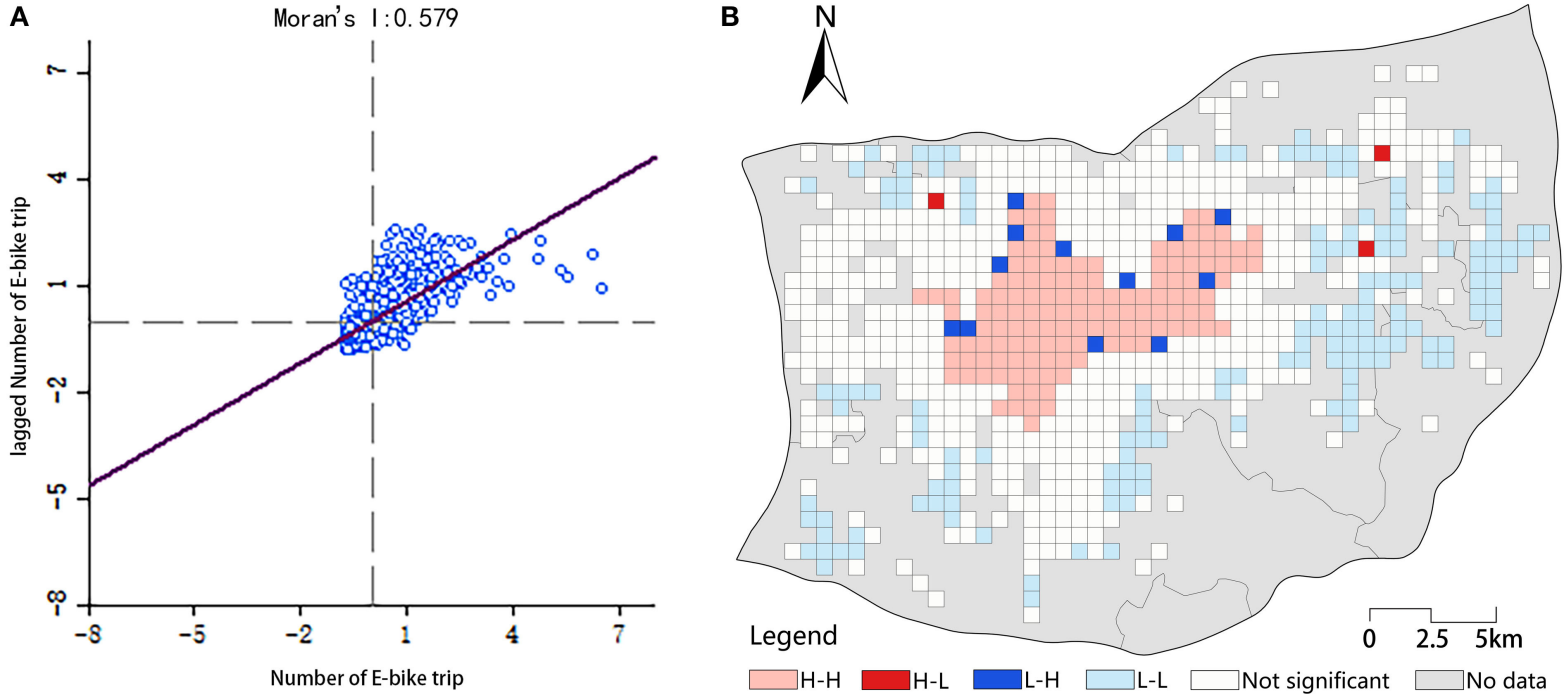
Spatial distribution pattern. **(A)** Moran scatterplot. **(B)** Local Moran's I clusters of e-bike trips.

The results of global Moran's I were presented in Figure 4A. There was a significant spatial autocorrelation (Moran's I = 0.579 and *p* < 0.001) in the distribution of e-bike usage in the study area.

Figure 4B presented the results of local Moran's I of e-bike trips. The results of LISA specifically reflected the local spatial correlation of e-bike travel volume. H-H clusters were concentrated in the central part of study area, which is a densely populated area with plenty of shopping areas. H-H areas were considered as the core development area of Jinan. Meanwhile, L-L clusters were distributed at the edge of the study area, which generally characterized by poor infrastructure configuration, complex terrain features, and relatively low population density. We also noticed an aggregated trend in the H-H and L-L units, respectively. In addition, L-H units were distributed in a fragmented form at the edge of the H-H units, while H-L units occurred randomly within the study area.

### Regression results of OLS model

The association between built environment and e-bike travel was estimated by the OLS model (Table 3). The adjusted R^2^ of OLS model is 0.428, which indicated that our model could explain 42.8% of the variation in e-bike trips in all grids. The VIF of the explanatory factor was <4, so there was no multicollinearity problem. The results showed that the four explanatory variables, i.e., eye-level greenery, building floor area, road density, and public service POI significantly promoted the e-bike trips, while the open sky index and NDVI significantly decreased e-bike trips. The relationships between land-use mix, commercial POI, distance to bus stop and e-bike trips were insignificant. However, the results did not indicate that these variables were not associated with e-bike trips, as the OLS model only calculates the overall effect of the study area.

**Table 3:** Results of ordinary least squares (OLS) model of built environment and e-bike (fishnet = 600 × 600 m, N = 770).

**Variable**	**Coef**.	***p***	**Std. error**	**VIF**
**Micro-scale built environment**
Eye-level greenery	0.106	0.001^**^	0.031	1.264
Open sky index	−0.137	0.000^***^	0.030	1.189
**Macro-scale built environment**				
Building floor area	0.148	0.000^***^	0.038	1.955
Land-use mix	0.009	0.711	0.033	1.424
Road density	0.133	0.000^***^	0.034	1.542
Commercial POI	0.054	0.229	0.045	2.707
Public service POI	0.329	0.000^***^	0.045	2.731
Distance to bus stop	0.027	0.399	0.032	1.396
NDVI	−0.149	0.000^***^	0.036	1.786
Adjusted R^2^	0.428
Residual sum of squares	435.517
Log-likelihood	−873.188
AICc	1,768.724

The numbers in parentheses represent p-values.

^**^ and ^***^ give the significance at the 5% and 1% levels respectively.

### Regression results of GWR model

The results of Moran's I revealed that there was significant spatial autocorrelation of e-bike usage, and the spatial heterogeneity of drive factors could not be revealed by OLS model. Therefore, we used the GWR model to further explore the association between built environment and e-bike usage. We applied MGWR software (version 2.2.1) for GWR model estimation (Table 4). The results demonstrated that the AICc of the GWR model was 1,559.603, which was about 12.1% smaller than the AICc of the OLS model. In addition, the adjusted R^2^ of the GWR model improved from 0.428 to 0.646, indicating that the GWR model had a better explanation of e-bike variation. Therefore, it could be illustrated that the fitting results of the GWR model were better than those of the OLS model.

**Table 4:** Results of geographically weighted regression (GWR) model of built environment and e-bike (fishnet = 600 × 600 m, *N* = 770).

**Variables**	**Min**	**1st quartile**	**Mean**	**3rd quartile**	**Max**	**STD**
**Micro-scale built environment**
Vegetation	−1.088	−0.016	0.049	0.177	0.410	0.216
Sky	−2.070	−0.230	−0.204	−0.005	0.170	0.362
**Macro-scale built environment**
Building floor area	−1.045	−0.003	0.089	0.235	0.547	0.271
Land-use mix	−1.195	−0.075	−0.032	0.066	0.481	0.196
Road density	−0.175	0.049	0.112	0.162	0.423	0.103
Commercial POI	−0.404	−0.157	−0.056	0.059	0.230	0.139
Public service POI	−0.131	0.111	0.271	0.405	0.905	0.250
Distance to bus stop	−1.034	−0.034	0.027	0.099	0.770	0.203
NDVI	−1.553	−0.328	−0.212	−0.030	0.074	0.270
Adjusted R^2^	0.646
Residual sum of squares	226.044
Log-likelihood	−620.704
AICc	1,559.603

The positive parameter estimates denoted the independent variable had positive impact on e-bike travel, and vice versa. The statistical results of GWR model were presented in Table 4, the coefficients of all explanatory variables had a wide range interval, which implied that the impact of built environment factors on e-bike travel was diverse in different spatial units. The mean values of the coefficients of the land-use mix and commercial POI variables in the GWR model (−0.032 and −0.056) differed significantly from their coefficient values in the OLS model (0.009 and 0.054), suggesting that the two factors had a stronger positive driving influence on e-bike usage in certain units. In addition, the open sky index had the largest standard deviation (STD = 0.362), which implied a large spatial variation in explaining the degree of association between the open sky index and e-bike trips. Moreover, majority units of road density and public service POI had positive local estimation parameters (1st Quartile>0); while the open sky index and NDVI had a higher number of negative local estimation parameters (3rd Quartile<0).

**Figure 5:**
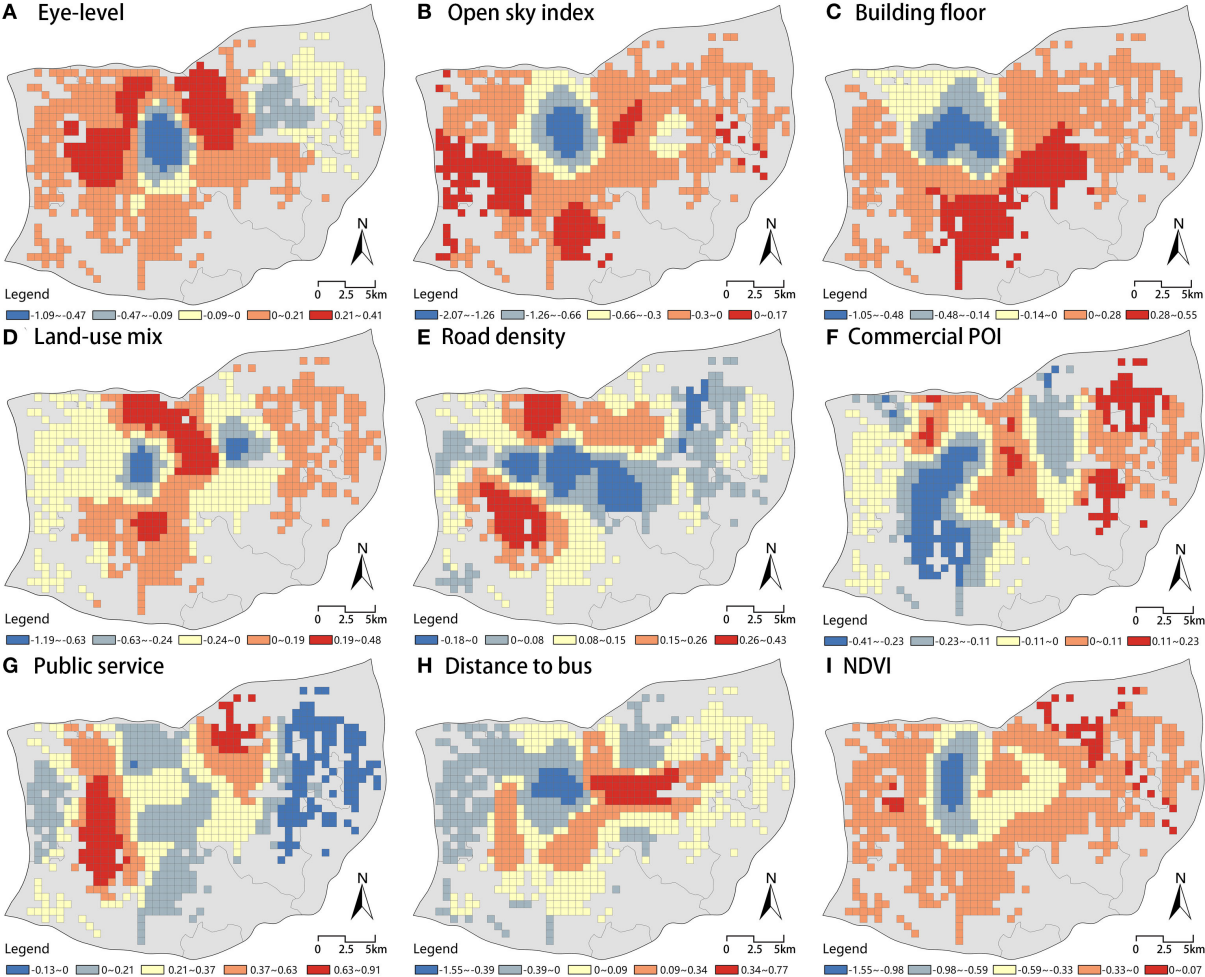
Spatial pattern of coefficients in different models. **(A)** Eye-level greenery. **(B)** Open sky index. **(C)** Building floor area. **(D)** Land-use mix. **(E)** Road density. **(F)** Commercial POI. **(G)** Public service POI. **(H)** Distance to bus stop. **(I)** NDVI.

The above statistical data analysis formulated a preliminary knowledge of the results of GWR model. ArcGIS was used to map the variable coefficient data of GWR model to realize the spatial visualization of the local coefficient values, which is convenient for us to deeply study the factors affecting e-bike travel from a spatial perspective. The spatial distribution of the local coefficients of 9 variables was presented in Figure 5, and the following conclusions can be drawn.

As shown in Figures 5B,I, open sky index and NDVI variables had significantly negative effects on e-bike usage in all grids. The positive values of the open sky index coefficient were aggregated in the southwestern part of the study area, where there were rolling hills and undulating terrain. In contrast to the open sky index, the positive values of NDVI coefficient were not densely distributed but fragmented in the eastern part of the study area. The lowest values of both coefficients were concentrated in the middle of the study area where belonged to the old city of Jinan with high building density and a large number of tourist attractions.

As shown in Figure 5G, the public service POI variable had globally a significant positive effect on e-bike trips. The coefficients of public service POI variable demonstrated a vertical band distribution, with an obvious demarcation line between positive and negative values. The positive coefficients in the central-eastern and western indicated that the public service POI variable laid a positive influence on e-bike usage in these regions; the negative coefficients of public service POI were concentrated in the eastern periphery of the study area, which was a developing area in Jinan, with an incomplete public service support facility.

As shown in Figures 5A,C,H, partially identical patterns in the coefficient distributions of the three variables of eye-level greenery, building floor area, and distance to bus stop were present. It was found that all three variables had both positive and negative effects on e-bike usage, and the proportions of units with positive and negative values were similar. In addition, the distribution of coefficients for all three variables were found to be concentric circles, expanding outward from the core area of maximum negative value in the middle with the coefficients progressively larger. The distribution of the positive units of the three variables differs. In the northwestern and northeastern part, eye-level greenery positively contributed to e-bike usage, while in the southeastern part, e-bike usage was positive associated with building floor area. The maximum positive value of distance to bus stop variable met the eastern side of the negative core and the distribution was demonstrated in east-west direction which was stripe-like.

The coefficients of land-use mix, road density and commercial POI were distributed differently as shown in Figures 5D–F. The coefficients of land-use mix were distributed in a binomial pattern, and the coefficients of the core area had the lowest negative values, and the positive values were distributed in the central and eastern part of the study area. In the central part, negative coefficients of road density were distributed from west to east, and the distribution pattern was same as the direction and location of the most important traffic artery in Jinan (Jingshi Road). The positive-coefficient units of commercial POI overlapped with several important commercial areas of Jinan, indicating that commercial facilities had a strong attraction and could promote e-bikes usage to some extent.

### E-bike travel aggregation characteristics

In Sections Spatial distribution pattern of e-bike travel volume and Regression results of GWR model, we investigated the local spatial effects of e-bike trips and the spatial distribution of the coefficients of each built environment variable by estimating local Moran's I and GWR model, respectively. We found a clear spatial aggregation of e-bike trips. To further investigate the connection between this phenomenon and the built environment, we calculated the mean coefficient values of each explanatory variable of the four types of spatial units (i.e., H-H, H-L, L-H, L-L) from Figure 3B to explore the built environment characteristics corresponding to four spatial types (63).

**Figure 6:**
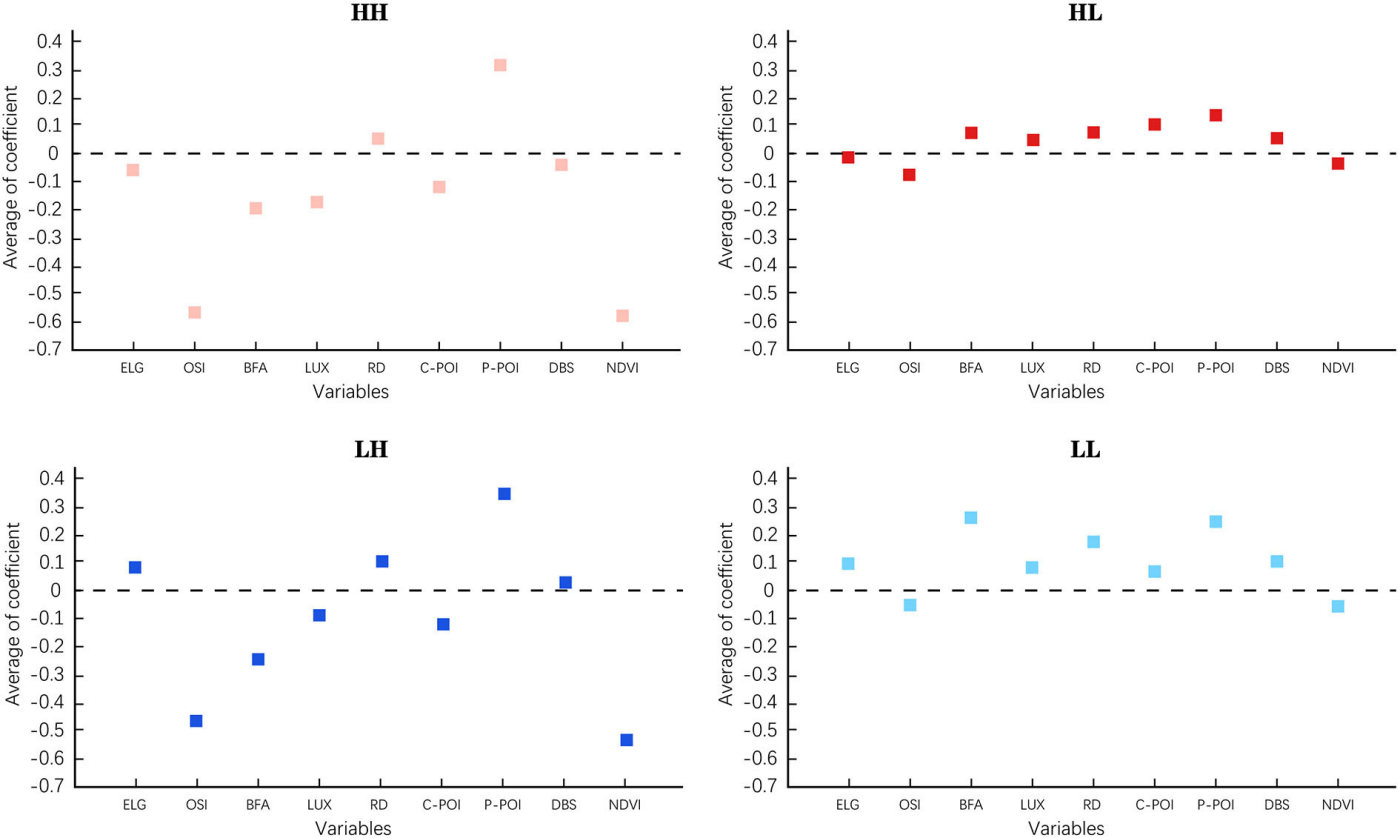
Average of built environment coefficient (ELG, Eye-level greenery; OSI, Open sky index; BFA, Building floor area; LUX, Land-use mix; RD, Road density; C-POI, Commercial POI; P-POI, Public service POI; DBS, Distance to bus stop).

Figure 6 depicted the results. The positive and negative of the ordinate mean coefficient represented the influence of built environment factors on e-bike travel; the positive value represented the promotion effect, and the negative value represented the prohibitive effect. The average coefficient value of the built environment factor indicated the influence degree of this factor on e-bike travel. The larger absolute value denoted the greater influence degree. The closer the absolute value to 0 indicated faint influence of this factor on e-bike travel. H-H cluster and L-H cluster had similar numerical distribution pattern, and the same pattern was found between L-L and H-L clusters. In addition, H-H and L-H clusters formed the units with large e-bike trips, while L-L cluster and H-L cluster represented the units with few e-bike trips. In the areas with large e-bike trips, the average coefficients of public service POI, open sky and NDVI had the largest absolute values, which indicated that these three factors had the strongest impact on e-bike trips, while other factors had relatively weak impact on e-bike trips. In the areas with few e-bike travel volume, the average absolute values of the coefficients of all types of built environment factors were close, and there was no significant difference among the coefficient values of all variables.

This section might serve as a reference for urban planners in decision making. For example, when creating an e-bike friendly area, priority can be given to build improved public services, reduce sky visibility to achieve the goal of promoting e-bikes usage. A profound understanding of the built environment influence can help the government to improve money efficiency and lay a solid foundation for building a cycling-friendly city.

## Discussion

### Major findings

As a new mode of active travel, e-bikes are widely accepted and used worldwide and are even considered to be able to replace motor vehicles in China (20). The healthful and low-carbon advantages of e-bikes are in line with the common consensus on low-carbon and healthy cities. Understanding the association between the urban built environment elements and e-bike travel is critical for urban planners. Although there are plentiful academic studies on the correlation between the urban built environment and various modes of transportation, few study focuses on the built environment effects on e-bike travel, especially from a global and local level. Moreover, previous empirical studies on built environment usually selected macro-level factors, ignoring the micro-level factors which involve residents' perception. This study addresses the abovementioned gap by unveiling the spatial relationship between built environment and e-bike travel of Jinan, and our study yields two major findings.

First, the relationship varies between different built environment variables and e-bike travel. The results of the OLS model indicate that road density is positively associated with e-bike usage. A well-connected street network increases the destination accessibility, thus encouraging e-bike usage. Pronounced diversity and proximity to bus stops reduce trip distance and offer a variety of possible transportation options for the residents (26, 69). In addition, various categories of destination accessibility have various correlations to e-bike trips. Public service POI has a significant positive relationship with e-bike trips, while commercial POI yields a negative relationship. Our finding is opposite of previous walking-related empirical studies, which prove public service POI has a significant negative impact on pedestrian volume and commercial POI has a positive impact (58, 70). The possible explanation is that the parking facilities for e-bikes are not well configured in most of the commercial establishments in Jinan. In addition, commercial areas attract a large number of people and are prone to block traffic, thus inhibiting e-bike trips. At the micro level, open sky has some inhibitory effect on e-bike usage. The eye-level greenery shows a positive correlation with e-bike usage while the NDVI variable shows a negative correlation with it. The results indicate a mismatch between the human street greenery perception and the bird's view greenery obtained through remote sensing satellites, which is consistent with previous study (61).

Second, there is significant spatial heterogeneity in the relationship between various built environment factors and e-bike usage. The distribution diagram of the GWR model coefficient values (Figure 5) indicates that the coefficients of most variables, except for public service POI, show a negative clustering at the center of the study area. We believe one reason for this phenomenon is that the planning of the old urban area is mainly centered on the preservation of historic sites, streets, and buildings, which has resulted in insufficient space to allocate e-bike related support facilities, including charging posts, parking areas and carriageways. Moreover, there is narrow spatial scale of residential areas in old downtown, thus e-bike travel can easily conflict with other modes of transportation (71, 72). The other reason to explain the finding is that large commercial complexes and scenic spots are not suitable for e-bike travel (73). Unlike small and medium-sized commercial facilities, large commercial facilities are more friendly for walking and motorized access because of the dense crowds and complex traffic conditions, while the high speed of e-bikes poses a greater safety risk in these areas. In addition, insufficiency in e-bike parking facilities in large commercial complexes and scenic spots act as a drag for e-bike riding (32). The old downtown of Jinan has a well-developed public transportation network which motivates residents to carry out their daily travel activities by walking and public transportation rather than using e-bikes (74).

### Planning implications

Jinan, as well as many other Chinese cities, is expanding in the rapid-urbanization context, which is a challenge for promoting e-bike commuting. This study indicates that when planning and building new urban areas, planners can achieve the purpose of regulating e-bike flows through the flexible settings of local built environment. In addition, interventions in the built environment can also alleviate traffic congestion in old urban areas to some extent. For example, in the areas with intensive e-bike travel congestion, increasing the sky openness by controlling the height of buildings and the number of trees can reduce the e-bike usages. On the contrast, in the areas without intensive travel congestion, increasing the greenery and constructing public service facilities along the roads can attract more e-bikes pass through these areas. Moreover, e-bike facility policies must also be integrated with vehicle traffic management policies, for example, setting speed limits, subsidizing the use of low-pollution vehicles, and constructing related supporting facilities. E-bike facility policies are also closely related to social equity and environmental justice. Chinese cities urgently need to develop long-term policies aimed at building cycling-friendly cities before they are permanently dominated by motorized forms of travel.

This study provides an effective decision support framework for policymakers to identify the most influential built environment factors associated with e-bike travel so as to build a healthy and low-carbon city (75). Based on this framework, tailored policy and planning interventions may enable residents to have a better e-bike travel experience in their local urban environment. In summary, planners and policymakers should take into fully account the positive or negative effects of different built environment elements on the e-bike usage and provide tailored planning strategy when carrying out specific e-bike related planning.

### Limitations

Despite of the theoretical and practical implications for future urban planning and public policy formulation, limitations of our study should also be acknowledged. First, OLS and GWR models are linear regression models, which involve only linear interpolation and have some limitations (37). Therefore, the relationship between urban built environment and e-bike usage should be further explored by model improvement. Second, the spatial scale effect and modifiable areal unit problem (MAUP) is the most important issues in urban planning and geography (76). Due to the difference in travel distance per unit time for choosing different travel modes, the effect of choosing different grid sizes as the basic unit of study may be sensitive to the results (53). In future research, the scale effect of built environment on e-bike usage could be further explored by transforming the grid size in the spatial dimension. Third, due to the limitations of data and technology, this paper only considered eye-level greenery and open sky as representatives of micro-level built environment variables. Future research hopes to further explore the relationship between more micro built environment elements and e-bike usage. Finally, this study did not consider the longitudinal variation of e-bike usage, future studies may work on revealing the effects of built environment on e-bike travel in different time periods.

## Conclusion

This study is the first to reveal the relationship between the built environment and e-bike travel in Jinan. Specifically, we designed a framework of built environment variables at both macro and micro levels using multiple sources of data to better quantify the built environment and we applied the OLS and GWR models to compare the coefficients of each variable globally and locally. The characteristics of built environment corresponding to different aggregation pattern of e-bike trips are analyzed using the GWR model and local Moran's I. The results of our study indicate key factors that need to be considered in the planning stage to reduce congestion pressure on urban traffic. For example, public services facilities and reasonable road network density can encourage e-bike traveling, while open sky and NDVI have an inverse impact on e-bike use. However, the impacts may vary across city level and further research is needed to identify the specific impacts across different areas. With more evidence, it would be easier to generalize findings from one region to others and inform built environment planning in China and other developing countries.

## Data availability statement

The raw data supporting the conclusions of this article will be made available by the authors, without undue reservation.

## Ethics statement

Ethical review and approval were not required for the study on human participants in accordance with the local legislation and institutional requirements. Written informed consent for participation was not required for this study in accordance with the national legislation and the institutional requirements.

## Author contributions

YY: data collection, data curation, methodology, and writing-original draft. YJ: conceptualization, methodology, and writing-original draft. NQ: conceptualization, visualization, and writing-review and editing. HG: data collection and writing-review and editing. XH: supervision, methodology, and writing-review and editing. YG: supervision and writing-review and editing. All authors contributed to the article and approved the submitted version.

## Conflict of interest

The authors declare that the research was conducted in the absence of any commercial or financial relationships that could be construed as a potential conflict of interest.

## Publisher's note

All claims expressed in this article are solely those of the authors and do not necessarily represent those of their affiliated organizations, or those of the publisher, the editors and the reviewers. Any product that may be evaluated in this article, or claim that may be made by its manufacturer, is not guaranteed or endorsed by the publisher.
